# TiO_2_-Decorated MXenes for Efficient UV Light Photocatalysis: A Comparative Study of Few- and Multi-Layer Structures

**DOI:** 10.3390/molecules31111945

**Published:** 2026-06-03

**Authors:** Daniela Balbontín, Sana Munir, Maibelin Rosales, Roberto Villarroel, Adriana Blanco, Francisco Gracia, Andreas Rosenkranz, Rodrigo Espinoza-González

**Affiliations:** 1 Department of Chemical Engineering, Biotechnology and Materials, Faculty of Physical and Mathematical Sciences, Universidad de Chile, Av. Beauchef 851, Santiago 8370456, Chile; dbalbontin1996@gmail.com (D.B.); sanamunir285@gmail.com (S.M.); lblanco@ing.uchile.cl (A.B.); fgracia@ing.uchile.cl (F.G.); arosenkranz@ing.uchile.cl (A.R.); 2 ANID—Millenium Science Initiative, Millenium Nuclei of Advanced MXenes for Sustainable Applications (AMXSA), Santiago 8370415, Chile; 3 BCMaterials, Basque Center for Materials, Applications and Nanostructures, UPV/EHU Science Park, 48940 Leioa, Spain; maibelin.rosales@bcmaterials.net; 4 Departamento de Física, Facultad de Ciencias Naturales, Matemática y del Medio Ambiente, Universidad Tecnológica Metropolitana, Las Palmeras 3360, Ñuñoa, Santiago 7800003, Chile; r.villarroel@utem.cl

**Keywords:** 2D materials, MXenes, TiO_2_, dye degradation

## Abstract

Water contaminated by textile dyes is a tremendous risk to human health and the environment due to its toxic and carcinogenic nature, thus requiring advanced and efficient removal strategies. Therefore, this study aimed to investigate the photo-oxidation performance of few- and multi-layer Ti_3_C_2_T_x_ nanosheets (MXenes) decorated with TiO_2_ nanoparticles for methyl orange removal from synthetic solutions. The quantification of photogenerated hydroxyl radicals by fluorescence revealed much higher OH^•^ production for TiO_2_-decorated samples, especially for multi-layer MXene, in which it was 2.8 times higher than that of few-layer MXene. However, photocatalysis was morphology-controlled: despite lower OH^•^, the few-layer MXene achieved the highest dye conversion (~45% after 5 h), attributed to shorter charge migration distances and more accessible TiO_2_ active sites, enabling effective h^+^ and superoxide-driven pathways. Moreover, the detected -OH surface terminations verified on MXenes promoted a notable adsorption capacity, especially for the multi-layer samples (~31%) via interlayer trapping and H-bonding. Therefore, our results demonstrate that few-layer MXenes are promising candidates for the efficient removal of methyl orange and highlight the potential of TiO_2_-decorated MXenes as promising photocatalysts for environmental remediation.

## 1. Introduction

Water pollution has emerged as a global concern, primarily due to the discharge of domestic, agricultural, and industrial waste, coupled with population growth, excessive pesticide and fertilizer use, and ongoing urbanization. These factors have resulted in a notable shortage of accessible clean water [1,2]. Approximately 80% of the global population encounters challenges related to drinking-water consumption. Furthermore, approximately 3% of deaths can be attributed to poor water quality, inadequate sanitation, and insufficient hygiene [3]. In this regard, the release of dyes from the textile industry accounts for approximately 20% of global pollution [4]. The production of textiles requires between 120 and 180 L of water per kilogram of fabric, and approximately 15% of dyes are combined with textile waste in water for disposal [5]. These dyes pose potential risks to living organisms because of their toxicity, carcinogenicity, and mutagenicity [6,7].

Dye pollution greatly impacts aquatic ecosystems by altering the coloration of water bodies and impeding light penetration. It is important to note that due to their high solubility in water, contaminating dyes are challenging to remove using conventional methods, and their persistence in the respective ecosystems poses risks to both environmental and human health [8,9]. Photocatalysis has emerged as a sustainable and environmentally friendly technology that offers an effective approach for degrading organic pollutants under solar irradiation [3]. Among the various photocatalysts, titanium dioxide (TiO_2_) is preferred because of its high stability, cost-effectiveness, and photoactivity [10,11]. However, its practical application is limited by the rapid recombination of photogenerated charge carriers under irradiation [12].

Various materials, including g-C_3_N_4_ [13], reduced graphene oxide (rGO) [14], and MXenes [15], have been investigated for their potential applications in pollutant degradation. MXenes are a class of two-dimensional (2D) early transition metal carbide, nitride, and carbonitride that have emerged as promising candidates for photocatalytic applications because of their high electrical conductivity, tunable surface terminations, and excellent photocarrier mobility [16]. MXenes are composed of transition metal atoms (M) and carbon and/or nitrogen atoms (X), with functional groups serving as surface terminations (Tx). Consequently, their general formula is M_n+1_X_n_T_x_ [17].

To enhance dye degradation, MXenes have been functionalized to adjust the bandgap to slightly higher values, facilitating the formation of electron–hole pairs during irradiation [18]. In the field of photocatalysis, leveraging the unique structure and properties of MXenes, these materials have been effectively employed to form heterostructures with various semiconductors, such as ZnO/MXene [18], Fe_2_O_3_/MXene [19], Nb_2_O_5_/MXene [20], V_2_O_5_/MXene [19], and La/MXene [20]. The overall objective was to improve the involved charge separation, conductivity, and light absorption [21]. These composite materials exhibit a broad bandgap range, enabling the degradation of toxic compounds under visible or ultraviolet light by initiating surface redox reactions. Functionalized Ti_3_C_2_T_x_ tends to exhibit enhanced photoactivity, particularly in materials with an increased bandgap. A notable instance is the degradation of methyl orange (MO) using BaTiO_3_/TiO_2_, where barium titanate (BaTiO_3_) possesses a bandgap of 2.31 eV, thereby augmenting the bandgap of the composite [21].

Another semiconductor that has garnered great attention for combining with MXenes is TiO_2_. The underlying reasons are its remarkable photocatalytic activity and physicochemical properties. The integration of Ti_3_C_2_T_x_ within the composite is pivotal as it supports the structure and mitigates the recombination of electron–hole pairs, thereby enhancing the separation and migration of photogenerated carriers [10]. This can be achieved by the in situ formation of TiO_2_ through controlled oxidation, which allows for improved adhesion onto MXene flakes and promotes the interfacial transfer of charge carriers [22]. Nevertheless, to achieve the best photocatalytic efficiency, it is crucial that TiO_2_ remains in the anatase phase, as this phase prolongs the lifespan of the charge carriers without recombination [23].

Despite recent advancements, notable gaps persist in the functionalization and integration of few-layer (FL) and multi-layer (ML) MXenes in TiO_2_ photocatalysis. Previous research has predominantly focused on the synthesis of MXene/TiO_2_ composites using conventional chemical methods, whereas solvothermal techniques have been less explored in this context. Furthermore, the fundamental mechanisms of charge transfer and the role of interfacial synergies within these heterostructures have not been thoroughly investigated, thereby limiting their optimization for practical applications in dye degradation.

Therefore, this study presents a layer-dependent approach for synthesizing Ti_3_C_2_T_x_ from Ti_3_AlC_2_ precursors functionalized with TiO_2_ using a straightforward solvothermal oxidation technique. It examines the impact of synthesis parameters by specifically comparing the FL and ML structures. By investigating the structural and electronic interactions between FL- and ML-Ti_3_C_2_T_x_ and TiO_2_ nanoparticles, this study aims to enhance the photocatalytic degradation of dye pollutants under UV illumination. Our findings contribute to the development of advanced composite materials with the overall goal of designing more efficient and scalable photocatalytic systems, thereby advancing the field of environmental remediation.

## 2. Results

### 2.1. Microstructural Analysis

**Figure 1:**
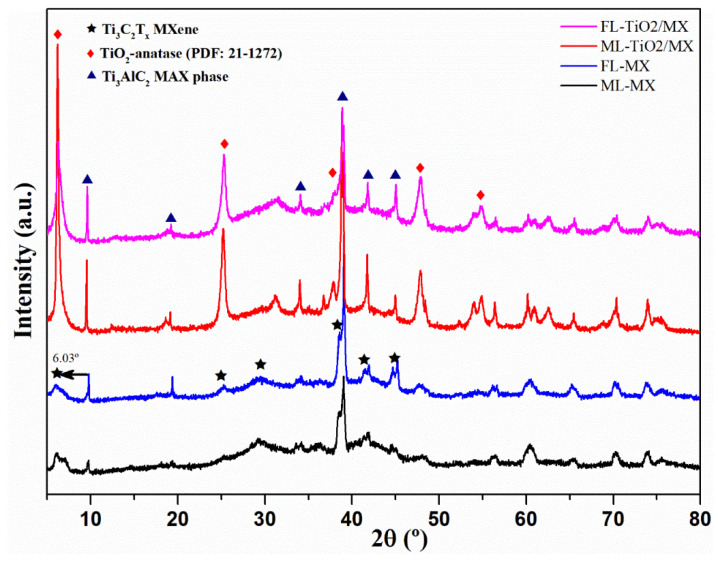
X-ray diffractograms of pure FL- and ML-MX and the corresponding FL-TiO_2_ and ML-TiO_2_ structures.

The X-ray diffractograms of the samples prepared in this study are shown in Figure 1. After the Ti_3_AlC_2_ MAX phase etching by in situ formation of HF, the preparation of ML- and FL-MX was observed as indicated by the shifting of the (002) peak from 9.43 to 6.03°. The shift and broadening of this peak are indicative of the removal of interlayer Al and the formation of Ti_3_C_2_T_x_, while the peak located at 38.9° indicates the presence of residual Ti_3_AlC_2_. This demonstrates the successful synthesis of Ti_3_C_2_T_x_ with a low amount of residual MAX phase. In contrast, after solvothermal treatment, a crystalline anatase TiO_2_ (JCPDS No. 21-1272) phase emerged in the ML-TiO_2_/MX and FL-TiO_2_/MX samples, confirming the decoration/functionalization of both MXenes, irrespective of the number of layers.

**Figure 2:**
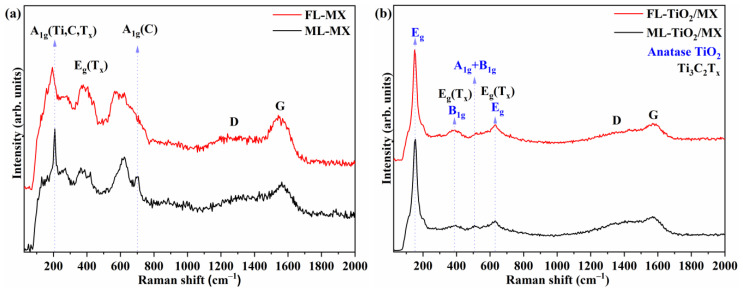
Raman spectra of all studied samples (**a**) prior to and (**b**) after the solvothermal treatment.

The layer-dependent structural features of MXenes can be inferred from Raman spectroscopy, as shown in Figure 2a, which depicts the Raman spectra of the ML- and FL-MX samples. The obtained spectra exhibited characteristic vibrational modes assigned to the D_3d_ symmetry of Ti_3_C_2_T_x_. According to Mulliken notation, the vibrations observed at ~200 and 700 cm^−1^ correspond to the out-of-plane A_1g_(Ti, C, Tx) and A_1g_(C) vibrations, respectively. Meanwhile, the broad contributions observed at ~280, 420, and 600 cm^−1^ belong to the E_g_ in-plane vibrational modes, which are strongly influenced by surface terminations (–O, –F, and –OH) [24,25]. In the 1300–1600 cm^−1^ region, the D (~1350 cm^−1^) and G (~1580 cm^−1^) bands arise from defect-induced carbon vibrations, serving as sensitive markers of MXene degradation into amorphous carbon [26].

The relative intensities, bandwidths, and frequency positions of the A_1_g and Eg modes are sensitive to interlayer coupling and stacking order, making Raman spectroscopy a powerful tool for distinguishing between FL- and ML-MXenes. In this regard, the red shift and decrease in intensity of the main A_1_g from 206 cm^−1^ in ML-MX to 195 cm^−1^ in FL-MX can be attributed to the progressive loss of interlayer interactions associated with the loss of stacking owing to the reduced number of layers [27]. Concurrently, the enhanced intensity of the E_g_ modes related to the terminal groups, observed in FL-MX, has also been reported as a characteristic feature of the increment of contributions of in-plane vibrations, indicating reduced stacking and FL configurations [28,29].

For comparison, Figure 2b shows the Raman spectra of the samples after the solvothermal process. In both cases, the vibrational modes associated with the anatase polymorph of TiO_2_ were predominant. The characteristic Eg stretching mode of anatase was clearly observed at ~150 cm^−1^, together with the bending modes (A_1g_, B_1g_) and the additional stretching mode located at approximately 400, 514, and 639 cm^−1^ [4]. Interestingly, both the formation of anatase and the emergence of amorphous carbon were more pronounced for the ML sample. In contrast, the Eg vibrational modes of the MXenes related to the surface terminations can still be distinguished in the FL sample.

**Figure 3:**
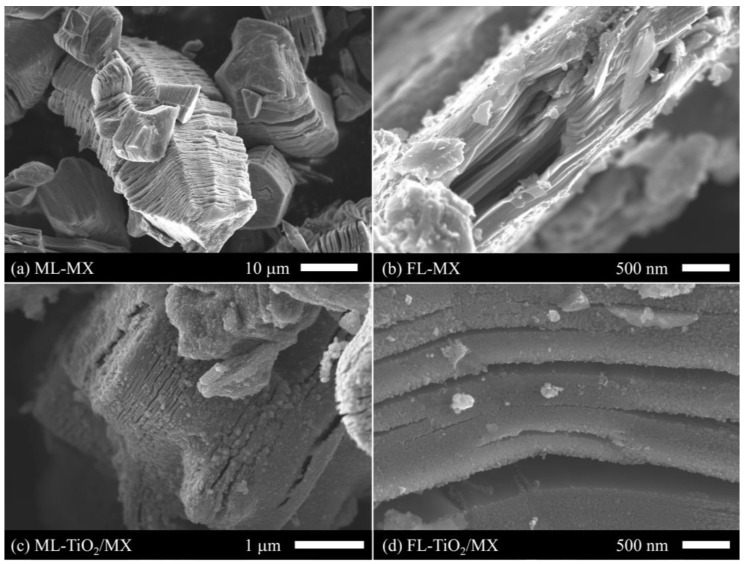
SEM micrographs of (**a**) ML- and (**b**) FL-MX and the resulting composite structures: (**c**) ML-TiO_2_/MX and (**d**) FL-TiO_2_/MX.

Representative SEM micrographs of the studied samples are shown in Figure 3. Figure 3a shows that the ML-MX sample exhibited particles of a layered MXene structure, in which every layer was a stack of several single layers of the 2D Ti_3_C_2_ structure. Meanwhile, Figure 3b presents the successful delamination of FL-MX based on the use of Li^+^ and H^+^ ions as intercalants, obtaining structures formed by a reduced number of layers. In contrast, the SEM images of the samples after the solvothermal treatment demonstrate that the laminar MXene structure is maintained, while the irregularities on their surfaces increase (Figure 3c,d). A granular structure was observed in both ML- and FL-MX due to the controlled oxidation, which was attributed to the TiO_2_ anatase phase, as confirmed by XRD.

**Figure 4:**
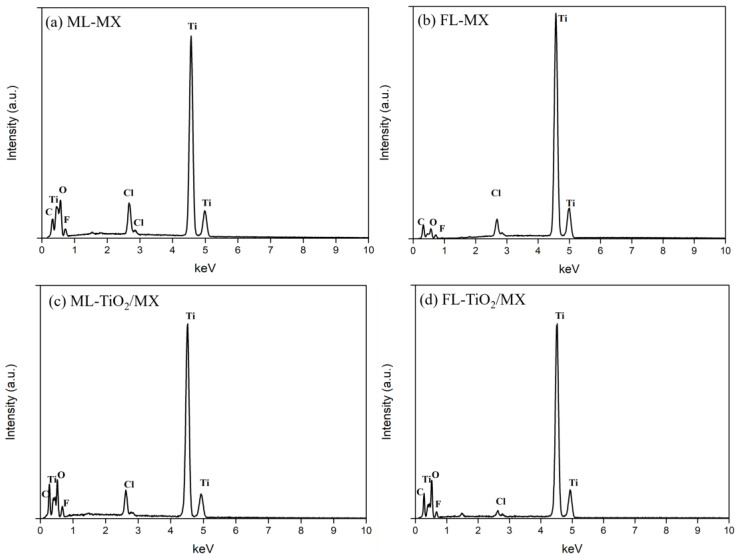
EDS analysis of (**a**) ML-MX and (**b**) FL-MX and the resulting composite structures: (**c**) ML-TiO_2_/MX and (**d**) FL-TiO_2_/MX.

The EDS results from the SEM measurements presented in Figure 4 prove the existence of Ti, C, O, F, and Cl in the ML-MX sample, which confirms the effective removal of Al from the MAX phase after etching (Figure 4a). The FL-MX sample exhibited a slight reduction in fluorine and chlorine content after delamination (Figure 4b). In addition, the results obtained by EDS, presented in Figure 4c,d, show a relative increase in the oxygen content for ML-TiO_2_/MX and FL-TiO_2_/MX, which can be associated with the formation of TiO_2_.

### 2.2. Quantification of OH^•^ Radicals

**Figure 5:**
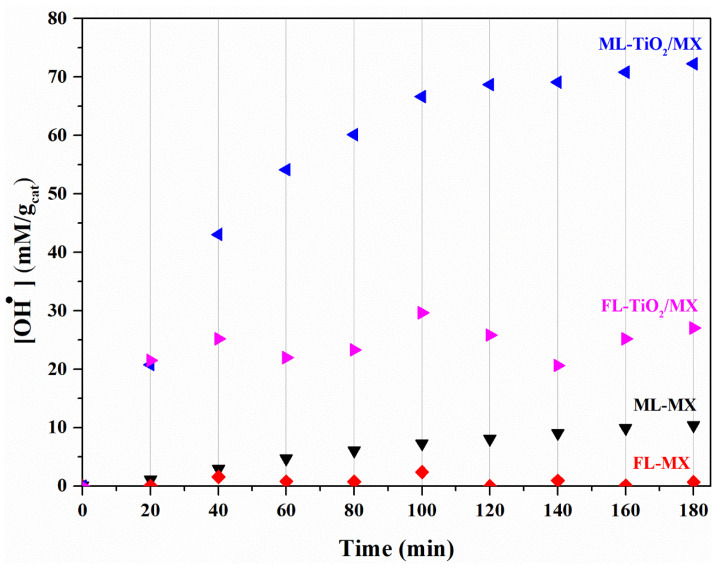
Concentration of hydroxyl radicals produced by the different samples under UV light irradiation.

Figure 5 shows the quantification of the photogenerated hydroxyl radicals by the synthesized samples during irradiation. The ML-MX sample exhibited slight OH^•^ radical production, whereas the FL-MX sample exhibited negligible photochemical activity under UV light irradiation. In contrast, the TiO_2_-decorated MXene samples demonstrated a markedly higher production of OH^•^ radicals, with ML-TiO_2_/MX material exceeding 70 μM/gcat after 180 min, which is almost 2.8 times greater than that produced by the FL-TiO_2_/MX sample under the same irradiation conditions. The superior generation of OH^•^ radicals observed for both FL- and ML-TiO_2_/MX composites is attributed to the synergistic interaction between the conductive Ti_3_C_2_T_x_ and the photoactive anatase TiO_2_ nanoparticles grown onto the 2D layers during the solvothermal treatment. Microstructural characterization confirmed that the in situ growth of TiO_2_ nanoparticles on the MXene surface resulted in a heterostructured interface, where TiO_2_ acted as the main active site that led to redox reactions under irradiation, while the 2D MXene provided excellent support for electron transport, thus helping to suppress electron–hole recombination [30]. Consequently, this interfacial interaction enhances charge separation, promoting increased participation in surface redox reactions and generating more reactive radicals that could enhance dye degradation. In contrast, the absence of TiO_2_ in FL- and ML-MX could be responsible for the slight or negligible OH^•^ production because the presence of photoactive sites is essential for initiating photochemical reactions. This suggests that pure MXenes are not photocatalytically active but boost an excellent charge transfer channel when coupled with semiconductors such as TiO_2_ [31]. Therefore, the FL- and ML-TiO_2_/MX systems benefit from the unique synergy between these conductive MXenes and TiO_2_, which promotes efficient charge separation and reduces carrier recombination rates.

### 2.3. Photocatalytic Performance by Methyl Orange Degradation

**Figure 6:**
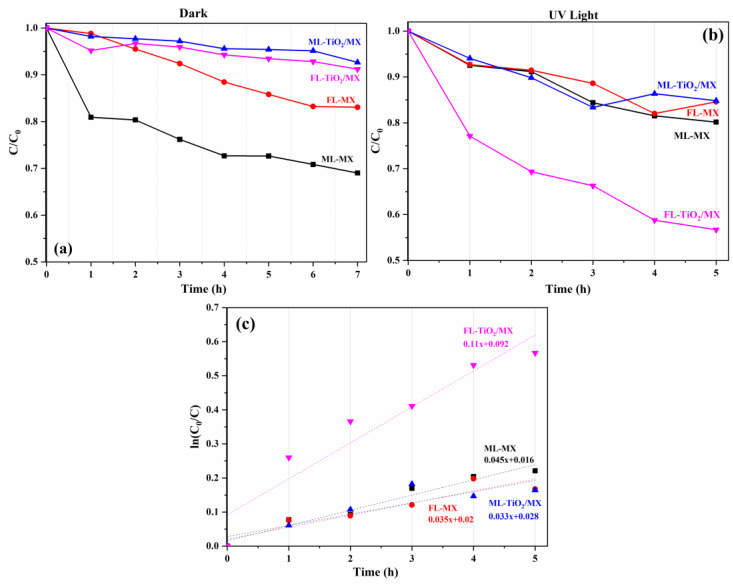
Comparative results of photocatalytic tests: (**a**) dark conditions; (**b**) UV light; (**c**) linear kinetic fit of MO degradation.

The performance of the materials in the degradation of methyl orange (MO) under dark conditions was evaluated to understand the effect of UV irradiation (Figure 6). In the first stage, the affinity of the synthesized materials for the dye under dark conditions was studied (Figure 6a). This figure reveals distinct trends across the four materials: ML-MX, ML-TiO_2_/MX, FL-MX, and FL-TiO_2_/MX. The ML-MX sample exhibited the highest MO adsorption capacity among the studied materials, capturing almost 31% of the dye. Given that both MO and Ti_3_C_2_T_x_ are negatively charged under the tested conditions, electrostatic interactions were unlikely to be the main driving force for adsorption. Instead, the observed affinity of MO for ML-MX can be attributed to hydrogen bonding between the surface -OH groups of Ti_3_C_2_T_x_ and the electronegative atoms of the MO molecule. Additionally, the ML morphology with an expanded interlayer spacing allows for the physical intercalation and trapping of MO molecules in the interlayer space. These interlayer spaces with larger available internal volumes could improve the adsorption sites compared to FL-structures. In contrast, both FL-MX and TiO_2_-decorated composites (FL-TiO_2_/MX and ML-TiO_2_/MX) demonstrated significantly reduced MO adsorption. This discrepancy can be attributed to differences in the surface properties, layer structure, and synergistic effects introduced by TiO_2_. Additionally, TiO_2_ reduces the accessibility of pollutants to the surface adsorption sites on Ti_3_C_2_T_x_. In the case of FL-MX, the reduction in the interlayer volume generates fewer sites for dye intercalation and, therefore, its uptake. In TiO_2_-functionalized composites, the TiO_2_ nanoparticles can partially cover the available MXene adsorption sites, such as the reduction of -OH groups due to the oxidation process, which physically blocks potential MO binding. Additionally, the formation of TiO_2_ nanoparticles during the solvothermal process may introduce a new surface composition with a different affinity for MO.

After confirming the high capacity of MXenes for dye adsorption, photocatalytic tests were performed under UV light. The results in Figure 6b demonstrate that after 5 h of exposure to UV light, the FL-TiO_2_/MX sample reached a dye conversion of approximately 45%, whereas the other samples were limited to less than 20% conversion, considering the remaining dye mass in solution after the adsorption phase.

The degradation percentages and kinetic parameters for all the samples are summarized in Table 1. The considerably greater activity of FL-TiO_2_/MX was estimated using the velocity constant (k_app_) obtained from the pseudo-first-order kinetic model (Figure 6c). In contrast, unoxidized Ti_3_C_2_T_x_ exhibited a high percentage of removal under dark conditions because of physical adsorption.

These results confirm that, although the presence of TiO_2_ on the photocatalyst surface reduced the adsorption capacity under dark conditions, it notably improved the dye degradation activity under UV light for the FL sample, which is consistent with the well-known UV-driven catalytic activity of TiO_2_.

**Figure 7:**
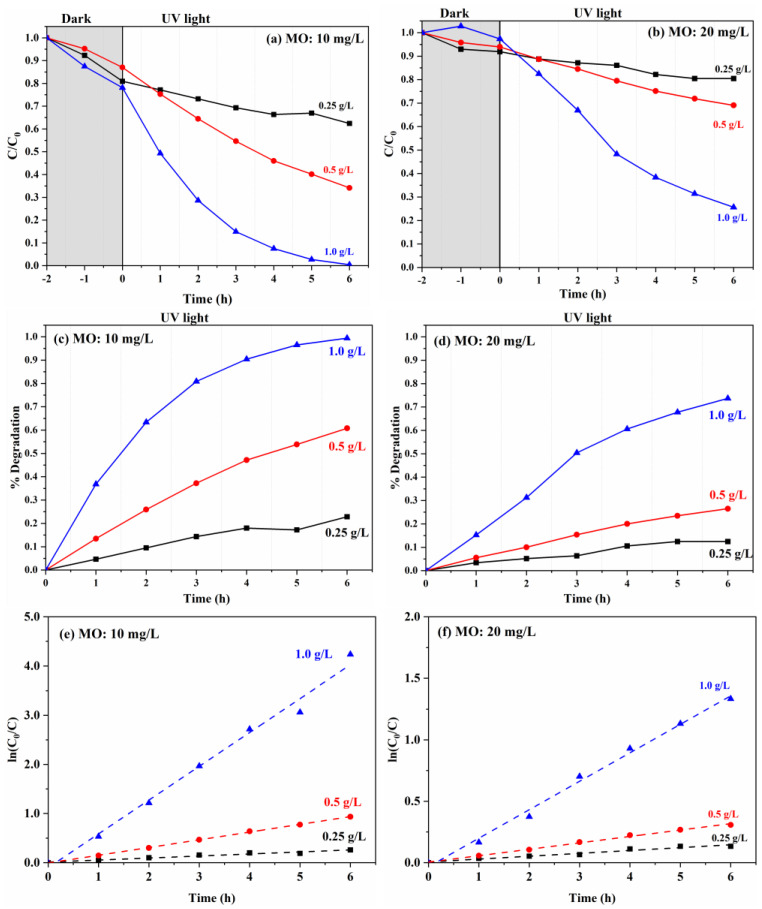
(**a**,**b**) Methyl orange degradation at different initial dye concentrations of (**a**) 10 mg/L and (**b**) 20 mg/L and different dosages of FL-TiO_2_/MX, respectively, considering 2 h of dye adsorption in dark conditions. (**c**,**d**) Photocatalytic activity at different initial dye concentrations of (**c**) 10 mg/L and (**d**) 20 mg/L and different amounts of FL-TiO_2_/Ti_3_C_2_T_x_, respectively. (**e**,**f**) Linear fitting for (**e**) 10 mg/L and (**f**) 20 mg/L methyl orange degradation using different FL-TiO_2_/MX doses.

Considering the best activity among the different samples, FL-TiO_2_/MX exhibited lower activity than previously reported materials [32]. This can probably be attributed to the reduced catalyst loading relative to the dye concentration. To determine the optimal catalyst dose and compare the FL-TiO_2_/Ti_3_C_2_T_x_ sample with other reported TiO_2_/Ti_3_C_2_T_x_ photocatalysts, additional tests were performed with two dye concentrations for up to 6 h of dye degradation. Figure 7a,b summarizes the degradation results for the solutions with MO concentrations of 10 mg/L and 20 mg/L, respectively. In both cases, three different catalyst loadings were considered: 0.25 g/L (initial condition), 0.5 g/L, and 1.0 g/L. After two hours of dye adsorption under dark conditions, higher degradation occurred at a catalyst loading of 1.0 g/L and a dye concentration of 10 mg/L. Under these conditions, the capture percentage under dark conditions after 2 h was 17.9%, and the degradation activity under UV light was 99.4% of the remaining dye after 6 h of exposure. When decreasing the catalyst loading to 0.25 g/L, the dye removal decreased to 22.9%. The same trend was observed when the dye concentration was increased to 20 mg/L. Consequently, it can be inferred that the higher the catalyst loading, the higher the dye degradation rate. Notably, at a concentration of 1.0 g/L, the degradation rate reached only 73.7%, implying that longer irradiation times are required for complete removal.

To complete the kinetic analysis, the results were recalculated as degradation percentages (Figure 7c,d) and fitted to a pseudo-first-order reaction rate. Figure 7e,f present the results of the linear kinetic fit of ln(C_0_/C) for each catalyst loading and both dye concentrations. The values calculated for the kinetic constant at a concentration of 10 mg/L are summarized in Table 2. The highest k_app_ value occurred at a catalyst dosage of 1.0 g/L, consistent with the higher amount of TiO_2_, given the higher amount of catalyst in the system. Moreover, the kinetic constant is 3.8 times lower than that reported previously by Li et al. [33] for a similar material, confirming that the catalysts prepared in this study contained a lower amount of the photoactive species.

Our results confirm the benefits of combining the photocatalytic effect of TiO_2_ with charge recombination attenuation owing to its close contact with the MXene surface. Bilal et al. also demonstrated a rapid MO degradation (less than 1 h) using a TiO_2_/Ti_3_C_2_ composite prepared by electrostatic self-assembly [34]. Although our results are somewhat slower under similar experimental conditions, the catalyst presented in this study possesses a considerably lower amount of TiO_2_. In this regard, the co-catalyst prepared by Li et al. [33] presented 3% of Ti_3_C_2_, which provides the material with a higher proportion of TiO_2_ in its composition, enhancing the photocatalytic behavior of the composites owing to the properties of Ti_3_C_2_ and anatase TiO_2_. Despite this, the formation of TiO_2_ via solvothermal oxidation generates a stronger interfacial interaction compared to self-assembly, which is expected to result in higher photocatalytic performance when the ratio TiO_2_/Ti_3_C_2_ is the same.

## 3. Discussion

Photocatalysis involves the generation of reactive oxygen species (ROS) by a photoactive material after photon absorption. ROS participate in redox reactions to degrade pollutants, and OH^•^ radicals are not the only reactive species involved in this degradation process. Instead, multiple parallel photocatalytic pathways, induced by the contribution of different reactive species, which strongly depend on the morphology, interfacial charge transfer efficiency, and accessibility of active sites, can affect the reaction kinetics. Although the terephthalic acid test quantifies the generated OH^•^, it does not directly measure the effectiveness of the interaction between these radicals and the adsorbed dye molecules. Overall, in ML structures such as ML-TiO_2_/MX, internal recombination, diffusion limitations, and restricted dye accessibility can reduce the effective participation of OH^•^ with MO molecules, thereby affecting its degradation [5]. Additionally, owing to its structure, the FL-TiO_2_/MX sample would exhibit shorter charge migration distances and stronger TiO_2_-MXene interfacial coupling, favoring the direct oxidation of MO by holes (h^+^) in the valence band and other ROS, such as superoxide anions ($O_{2}^{•-}$), as reported by Peng et al. [21]. Consequently, the accessibility of MO would be controlled by the morphology of these materials. A possible explanation is that in ML-TiO_2_/MX, the TiO_2_ and generated radicals are mainly located inside the piled multi-layers, with limited dye diffusion into the internal layers. In contrast, in FL-TiO_2_/MX, the active sites would be more accessible to the surface, facilitating immediate contact between the adsorbed MO and h^+^, $O_{2}^{•-}$, and OH^•^ for MO degradation [35]. This would explain why FL-TiO_2_/MX achieved a higher degradation compared to ML-TiO_2_/MX despite the reduced OH^•^ concentration measured. In this regard, MO degradation arises from more efficient charge transport and utilization in redox reactions, enhanced direct hole oxidation, and the participation of other radicals, such as superoxide anions, rather than from OH^•^ radical concentration alone.

**Figure 8:**
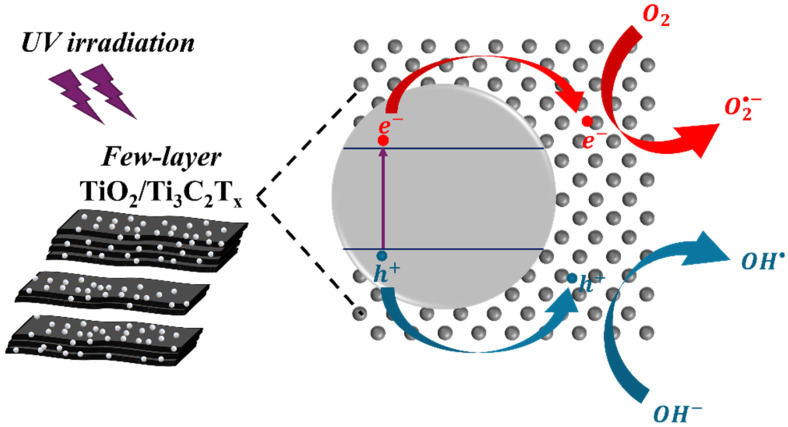
Schematic illustration of the derived dye degradation mechanism based on photocatalysis using FL-TiO_2_/MX.

As depicted in Figure 8, the formation of anatase nanoparticles on the TiO_2_/Ti_3_C_2_T_x_ samples notably enhanced the photocatalytic activity by increasing the generation of photoinduced charge carriers. Upon UV irradiation, TiO_2_ nanoparticles absorb photons to generate electron–hole pairs, where electrons can be effectively transferred to the Ti_3_C_2_T_x_ surface, which acts as an electron reservoir owing to its high conductivity and metallic nature [30].

The interfacial charge separation reduces the recombination rate of electron–hole pairs, thereby prolonging carrier lifetimes and improving surface redox reactions. The reactions associated with dye photocatalytic degradation are proposed [36]:

(1) $$TiO_{2}+h\nu \to TiO_{2}\left(e^{-}+h^{+}\right)$$

(2) $$TiO_{2}\left(e^{-}+h^{+}\right)+Ti_{3}C_{2}T_{x}\to TiO_{2}\left(h^{+}\right)+Ti_{3}C_{2}T_{x}\left(e^{-}\right)$$

(3) $$TiO_{2}\left(h^{+}\right)+OH^{-}\to TiO_{2}+OH^{•}$$

(4) $$Ti_{3}C_{2}T_{x}\left(e^{-}\right)+O_{2}\to Ti_{3}C_{2}T_{x}+O_{2}^{•-}$$

(5) ROS+dye→degraded products

The strong oxidative potential of the holes in the TiO_2_ valence band promotes a reaction with adsorbed water or hydroxyl ions to produce hydroxyl radicals, which play a crucial role in the degradation of MO molecules. Simultaneously, the electrons transferred to the conduction band can participate in the reduction of molecular oxygen to produce superoxide radicals. ROS formation enhanced the photocatalytic degradation of MO compared to unoxidized Ti_3_C_2_T_x_, which lacks semiconducting photoactive sites. This enhanced behavior is supported by the photochemical characterization, which reflected an increase in hydroxyl radical production in TiO_2_-decorated MXene samples. Although pure Ti_3_C_2_T_x_ exhibits a high dye removal capability through adsorption processes without irradiation, these materials exhibit poor photocatalytic activity owing to the lack of photoactive sites required to drive the generation of OH^•^ under irradiation.

## 4. Materials and Methods

### 4.1. Synthesis of MXenes (Ti_3_C_2_T_x_)

MXenes were prepared by chemical etching of a high-purity Ti_3_AlC_2_ MAX phase purchased from Jilin 11 Technology Co., Ltd. (Changchun, China). Chemical etching was performed by in situ formation of hydrofluoric acid by mixing lithium fluoride (LiF Suprapur^®^, Merck 99.99%, Darmstardt, Germany) and hydrochloric acid (HCl Supelco^®^, Merck 32%, Darmstardt, Germany). The molar ratio of the chemical agents to the MAX phase was 1:7.5. LiF (0.5 g) and HCl (9 M, 5 mL of 9 M HCl) were mixed and stirred in a Teflon beaker at 300 rpm for 10 min. Subsequently, 0.5 g of the MAX-phase precursor was added to the solution at a rate of 100 mg/5 min. The mixture was placed in a glycerin bath at 50 °C for 24 h [37]. The solution was then centrifuged to achieve a pH of 6. The obtained liquid was then filtered under vacuum using a 47 mm diameter PTFE membrane purchased from Tisch Scientific (Cleves (OH), USA) with a pore size of 0.22 µm, which was previously moistened with technical-grade ethanol to allow the passage of water. Finally, the samples were dried in a vacuum oven (Daihan Labtech Co., Ltd., Namyangju-si, Republic of Korea) at 70 °C and −0.09 MPa for 24 h. The sample obtained via this route was labeled ML-MX.

To prepare FL-MXene, the as-prepared ML-MX was sonicated at 80 kHz in ice water for 1 h to delaminate the MXene flakes. The sample was then dried in a vacuum oven at 70 °C and −0.09 MPa for 24 h, from which the material labeled as FL-MX was obtained.

### 4.2. Preparation of TiO_2_/Ti_3_C_2_T_x_

**Figure 9:**
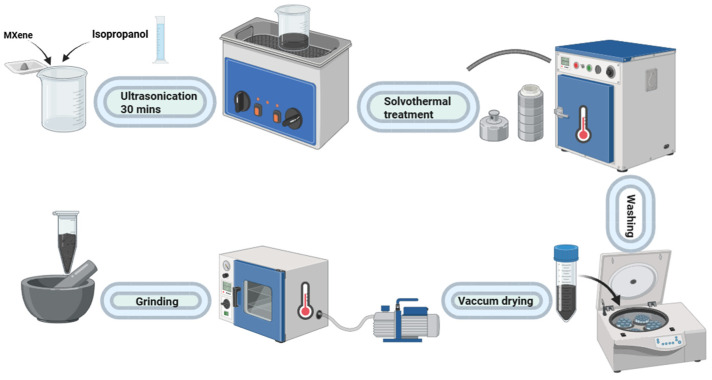
Schematic illustration of the processing steps necessary for TiO_2_/Ti_3_C_2_T_x_ generation.

To promote the formation of TiO_2_ on MXenes, 0.2 g of either the ML- or FL-MX was added to 16 mL of isopropanol and placed in an ultrasonic bath at 80 kHz for 30 min. The solution was then placed in a reactor and heated at 90 °C for 24 h, as shown in Figure 9 [38]. The solution was transferred to 50 mL Falcon tubes and centrifuged at 8000 rpm for 30 min. The samples were dried in a vacuum oven at 70 °C. The final material was ground using a piston mortar to obtain a powder. The samples prepared using this method were named ML-TiO_2_/MX and FL-TiO_2_/MX.

### 4.3. Microstructural Characterization

A Bruker D8 Advance Diffractometer (Karlsruhe, Germany) was used to perform X-ray diffraction (XRD) of the samples. Additionally, Raman spectroscopy was performed using a Raman Witec Alpha 300 spectrometer (Ulm, Germany) with a 532 nm laser for excitation to analyze the formation of MXenes and TiO_2_. The microstructures of all samples were studied using scanning electron microscopy (SEM) with a Thermo Fisher INSPECT-F50 (Eindhovenm, The Netherlands). This equipment was also used to measure the composition of the obtained MXenes using energy-dispersive X-ray spectroscopy (EDS).

### 4.4. Quantification of Hydroxyl Radicals

The OH^•^ radicals were quantified under irradiation via fluorescence spectroscopy using a PerkinElmer LS 55 Fluorescence Spectrometer (Waltham, MA, USA). Quantification was performed through the hydroxylation reaction of terephthalic acid (TA) using the hydroxylated and fluorescent product of this reaction (2-hydroxyterephthalic acid), according to a previously described method [39].

### 4.5. Photocatalytic Experiment

To perform the photocatalytic experiments, tests under dark conditions were first performed on the samples using a 20 mg/L MO solution, with a catalyst loading of 50 mg per 200 mL of dye (0.25 g/L), to determine the time required to reach adsorption equilibrium before irradiation. Although the TiO_2_/Ti_3_C_2_T_x_ composites and pure Ti_3_C_2_T_x_ reached adsorption equilibrium in approximately 2 and 12 h, respectively, all samples were subjected to a 15-h dark saturation period to ensure the same initial conditions prior to photo-irradiation.

Subsequently, for the experiments under UV light, the same dye concentration and catalyst loading were placed in a reactor with a jacket under continuous stirring, connected to the water flow to prevent evaporation, in an assembly consisting of a 25 × 25 × 40 cm^3^ acrylic box lined with an Interfilm © Sun-Gard Sheet (Santiago, Chile) with a 10 × 10 cm^2^ top outlet. A 115 V and 100 W 365 nm UV bulb without a visible light filter (100 mW/cm^2^ power irradiation) was used as a light source. The solution was stirred under exposure to the UV bulb for 5–6 h, and aliquots of the sample were collected every 1 h. Each aliquot was vacuum-filtered using a hydrophilic polyvinylidene fluoride (PVDF) membrane (Darmstardt, Germany) with a pore size of 0.22 μm, and the filtered liquid was subsequently recovered in a quartz cuvette. The decrease in the concentration of MO was quantified using a Jasco V-530 UV/Vis Spectrophotometer (Tokyo, Japan) at 465 nm, which is the maximum absorption wavelength of MO.

## 5. Conclusions

This study investigated the photocatalytic performance of FL- and ML Ti_3_C_2_T_x_ decorated with TiO_2_ nanoparticles for the degradation of MO dye under UV light irradiation. Ti_3_C_2_T_x_ was synthesized using in situ chemical etching, and solvothermal oxidation was used to produce TiO_2_ on the surface of the MXene layers, as evidenced by complementary material characterization. The photocatalytic activity was evaluated by measuring the degradation of MO dye and quantifying the generation of OH^•^. The results clearly demonstrate that FL-TiO_2_/MX exhibits the highest photocatalytic performance, achieving approximately 45% MO degradation after 5 h of UV light exposure. This enhanced activity was attributed to the synergistic interaction between the conductive Ti_3_C_2_T_x_ and the photoactive anatase TiO_2_, which promoted efficient charge separation and reduced carrier recombination rates. This study highlights the potential of TiO_2_-decorated MXenes as promising photocatalysts for environmental remediation applications.

## Figures and Tables

**Table 1:** Photocatalytic responses for MO degradation of all material systems studied.

Sample	Removal in Darkness Efficiency [%]	Degradation Efficiency [%]	k_app_	R^2^
ML-MX	30.98	19.83	0.045	0.97
FL-MX	16.93	15.42	0.035	0.91
ML-TiO_2_/MX	7.32	15.17	0.033	0.87
FL-TiO_2_/MX	8.78	43.28	0.106	0.97

**Table 2:** Kinetic constants k_app_ for different FL-TiO_2_/MX catalyst loadings. Dye concentration: 10 mg/L.

Catalyst Loading [g/L]	Overall Dye Removal Efficiency [%]	k_app_ [1/h]	R^2^
0.25	22.85	0.04	0.955
0.50	60.77	0.16	0.999
1.00	98.55	0.69	0.987

## Data Availability

Data are available upon request.
